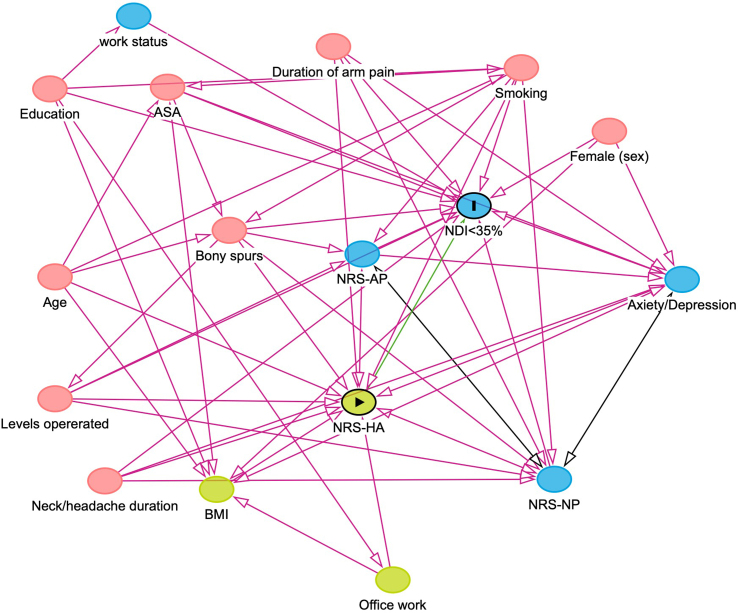# Preoperative headache severity and the risk of unsuccessful outcomes after anterior surgery for degenerative cervical radiculopathy: a population-based study from the Norwegian Registry for Spine Surgery

**DOI:** 10.1016/j.bas.2026.106100

**Published:** 2026-05-16

**Authors:** Samer Habiba, Elisabet Danielsen, Tor Ingebrigtsen, Tonje O. Johansen, Jarle Sundseth, Erling Myrseth, Roar Kloster, Øystein P. Nygaard, John-Anker Zwart, Tore K. Solberg

**Affiliations:** a Department of Neurosurgery, University Hospital of North Norway, Tromsø, Norway; b Department of clinical Medicine, UiT the Arctic University of Norway, Tromsø, Norway; c The Norwegian Registry for Spine Surgery (NORspine), University Hospital of North Norway, Tromsø, Norway; d Department of Neurosurgery, St.Olavs Hospital, Norway, Trondheim, Norway; e Department of Neurosurgery, Rikshospitalet, Oslo University Hospital, Norway; f Department of Research and Innovation, Division of Clinical Neuroscience, Oslo University Hospital, Norway; g Institute of Clinical Medicine, University of Oslo, Norway; h Department of Neurosurgery, Haukeland University Hospital, Bergen, Norway; i Norwegian National Network for Spinal Surgery, Norway

**Keywords:** Degenerative cervical radiculopathy, Headache, Prognostic factor, Neck disability index, Patient reported outcome measures

## Abstract

**Introduction:**

Degenerative cervical radiculopathy (DCR) is the most frequent indication for cervical spine surgery. Many patients presenting with arm pain and neck pain, also complain of headache (HA).

**Research question:**

This study investigates whether preoperative HA is an independent predictor of a non-successful surgical outcome.

**Materials and methods:**

A cohort of 6234 patients undergoing anterior cervical surgery for DCR were operated, prospectively registered in the Norwegian Registry for Spine Surgery (NORspine) and followed for one year. Baseline headache severity measured by numeric rating scale (NRS-HA) was used as exposure in multivariable mixed logistic regression models to determine its association with surgical non-success, defined as <35% improvement in the Neck Disability Index (NDI) at 12 months, while adjusting for possible confounders.

**Results:**

A total of 4689 (75%) reported DCR-concomitant headaches at baseline, and the loss to follow-up at 12 month was 30%. Each 1-unit increase on the NRS-HA scale was significantly associated with a higher likelihood of surgical non-success, as shown by both the multivariable regression model (8% increase per unit, odds ratio (OR: 1.08, 95% confidence interval (CI): 1.05-1.11, p < 0.001) and the mixed logistic model (21% increase per unit, OR: 1.21, 95% CI: 1.14-1.27, p < 0.001).

**Discussion and conclusion:**

Higher preoperative headache severity was an independent prognostic factor for non-successful outcomes following DCR surgery. This information can be used in preoperative counselling, aimed at improving patient selection and prognosis.

## Highlights

• Headache was common, with 75% of patients reporting degenerative cervical radiculopathy-concomitant headache (DCR-HA) preoperatively.
• Higher preoperative headache severity independently predicted surgical non-success at 12 months.
• For each 1-point increase in NRS-HA, the odds of non-success increased by 8% in the multivariable model (population level) and 21% in the mixed logistic model (individual level).
• Preoperative headache severity may be useful in patient counselling.

## 1 Introduction

Degenerative Cervical Radiculopathy (DCR) is the most common indication for cervical spine surgery (Carette and Fehlings, 2005). It is caused by nerve root impingement, leading to radicular arm pain, often accompanied by numbness and/or weakness. Consequently, outcome evaluations and prediction models tend to focus on improvement of arm pain-disability (Mjåset et al., 2020, 2022; Hebert et al., 2023). Still, many patients also complain of additional neck pain and headache. In particular headache, has received little attention, even if its prevalence (58–88%) in DCR patients seems to be similar to that of neck pain (Persson et al., 2007; Thind et al., 2020; Sun et al., 2015; Schrot et al., 2014; Zhou et al., 2024; Yang et al., 2022; Pang et al., 2020; Shimohata et al., 2017). This is probably because DCR concomitant headache (DCR-HA) alone is not recommended as indication for DCR surgery (Persson et al., 2007; Thind et al., 2020). However, the prognostic role of headache in surgical outcomes remains unclear and warrants further investigation. DCR-HA is not well defined in the literature and may indicate other underlying pain syndromes or primary headache disorders (Habiba et al., 2026). This means that although it tends to improve after surgery (Thind et al., 2020; Zhou et al., 2024; Habiba et al., 2026), headache severity could in fact be a predictor of unsuccessful outcomes measured by arm pain and disability.

Other known prognostic factors for unsuccessful outcomes are longer symptom duration, female sex, smoking, receiving sickness benefits, high body mass index, depression and/or anxiety, comorbidity, low education level (Hebert et al., 2023; Zhou et al., 2024; Wong et al., 2014; Khan et al., 2020; Peolsson and Peolsson, 2008; Jacob et al., 2022; Passias et al., 2018; Devin et al., 2021). Furthermore, factors as neck pain, spondylosis, arm pain and myelopathy are reported to be associated with headaches (Pang et al., 2020; Lin et al., 2018).

Evaluation of prognostic factors is crucial for informed shared decision making, patient selection to surgery, and for calibrating surgeons and patients’ expectations about outcomes. The aim of this study is to investigate if headache is an independent prognostic factor of non-success following DCR surgery.

## 2 Materials and methods

### 2.1 Study design

This study is a retrospective analysis of prospectively collected data, and it adheres to the principles of Prognosis Research Strategy (PROGRESS) framework II: Prognosis factor research (Riley et al., 2013). It has been reported in accordance with the Strengthening the Reporting of Observational Studies in Epidemiology (STROBE) statement. The study protocol is published at ClinicalTrials.gov identifier NCT06885216.

### 2.2 Setting and data source

**Fig. 1:**
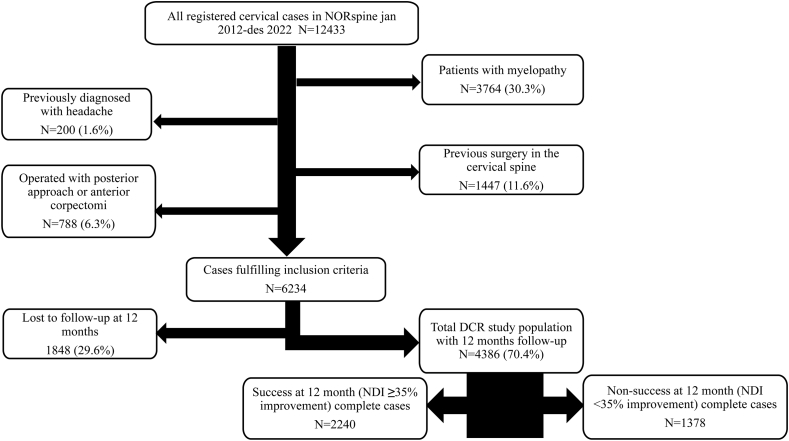
Study flow chart.

In this population-based national cohort study, we included 6234 consecutive patients operated for DCR in six public and six private hospitals (specialist health care) and followed them for 12 months. Data were retrieved from the Norwegian Registry for Spine Surgery (NORspine) for cases operated between January 2012 and December 2022 (Fig. 1). The NORspine is a degenerative registry and excludes cases operated for tumors, fractures, vascular issues, and primary infections. In 2021 the NORspine covered all clinics performing cervical spine surgery, capturing 81% of all patients operated for DCR across Norway (Solberg et al.). The DCR-diagnosis was based on a standard diagnostic work-up encompassing surgeons clinical and radiological evaluation.

### 2.3 Patient and public involvement

Two patient representatives have been involved in the planning and design of the study and will take part in the dissemination of results. In addition, a patient organization representative who is a member of NORspine's advisory board will assist in conveying results to patients.

### 2.4 Eligibility criteria

Patients aged 16 years or older undergoing surgery for DCR were eligible. Included procedures were single- or multilevel anterior cervical discectomy and fusion (ACDF), with or without an anterior plate, and artificial cervical disc replacement (ACDR). We excluded patients with prior cervical spine surgery; those treated with corpectomy or a posterior approach; those operated for degenerative cervical myelopathy (assessed by indication and/or completion of Ranawat score by the operating surgeon); and those with a preexisting headache diagnosis documented by the surgeon.

### 2.5 Data collection

At admission for surgery (baseline), patients completed a self-administered questionnaire, recording sociodemographic characteristics (age, sex, smoking status, education level, employment status), duration of symptoms, and patient-reported outcome measures (PROMs), including the numeric rating scale for arm pain (NRS-AP), neck pain (NRS-NP), and headache (NRS-HA); and the Neck Disability Index (NDI). Health-related quality of life was measured by the EuroQol 5-Dimension, 3-Level (EQ-5D-3L). Anxiety and/or depression was assessed according to the EQ-5D 5th item (no, moderate or severe problems). All PROMs were reassessed at 3 and 12 months postoperatively. Clinical data, surgical treatment and approach, diagnosis including indication for surgery (e.g., radiculopathy, radiological findings), comorbidities (such as a prior headache diagnosis), length of hospital stay, and perioperative complications, were documented by the attending physician or surgeon.

### 2.6 Outcome measurement

The primary outcome measure was non-success 12 months after surgery (yes/no), defined by an improvement of the NDI score of less than 35% at 12 months (Mjåset et al., 2020). This cut off has also been used in several RCTs, evaluating clinically meaningful treatment effects (Taso et al., 2025; de Vries et al., 2025). The NDI measures neck related disability in ten different domains: pain, personal care, lifting, reading, headaches, concentration, work, driving, sleeping and recreation, using a 6-point ordinal scale (0−5). The items are summarized and recalculated to a percentage score ranging from 0 to 100 (no to maximum disability). (Vernon, 2008).

### 2.7 Exposure variable

The severity of baseline headache was assessed by a numeric rating scale (NRS-HA) reporting pain on an 11-point scale from 0 to 10 (“no pain” to “worst conceivable pain”) (Haefeli and Elfering, 2006), by asking patients the following question: “How would you grade the pain you have had in your head during the last week?“.

### 2.8 Hypothesis

Null hypothesis (H0) Preoperative headache severity is not an independent prognostic factor of unsuccessful outcome after DCR surgery.

Alternative hypothesis (H1) Preoperative headache severity is an independent prognostic factor of unsuccessful outcome after DCR surgery.

### 2.9 Confounder criteria for inclusion in multivariable analysis

We identified candidate predictors a priori, i.e. guided by clinical judgement and clinical relevance reported in previous studies (Supplementary Table 1). (Hebert et al., 2023; Zhou et al., 2024; Wong et al., 2014; Khan et al., 2020; Peolsson and Peolsson, 2008; Jacob et al., 2022; Passias et al., 2018; Devin et al., 2021) Directed Acyclic Graphs (DAGs) were constructed for two conceptual frameworks: a “base” model and an extended “sociobiological model” (Supplementary Fig. 1–2). In a consensus meeting the study group used the DAGs to visually map assumed relationships between the exposure, outcome and potential confounders, being included in the multivariable analyses.

From the DAG, we derived a minimal sufficient adjustment set to estimate the direct effect of NRS-HA on NDI <35%.

We evaluated pairwise correlations among candidate predictors to assess collinearity. Duration of arm symptoms and NRS-AP were highly correlated (Spearman's rho = 0.70), and NRS-HA correlated with NRS-NP (rho = 0.52). To improve model stability and reduce multicollinearity, duration of arm symptoms and NRS-NP were excluded from the confounders to be included in the multivariable analysis for the “base” model.

We then expanded the DAG to incorporate work status, and education as possible confounders (Supplementary Fig. 2). Only education was eligible for additional inclusion in the multivariable model, yielding the extended “socio-biological” model, defined as the “base” model eligible confounders plus education. Conclusive results would be based on best model.

### 2.10 Sample size estimation

There are no closed-form formulas for sample size calculation in multivariable prognostic factor studies. Applying the 10 events-per-predictor rule of thumb, suggests a sample of 1000 with an expected 12% prevalence of non-successful outcomes would allow for the inclusion of 12 predictors, including the exposure. This suggests that our sample size by far exceeds the minimum requirement estimate for regression based prediction models (Riley et al., 2020).

### 2.11 Statistical analyses

Differences in proportions were analysed using chi-square tests. Mean differences within and between groups were assessed using paired and independent samples t-tests. Correlation coefficients (Spearman's or Pearson's) were calculated based on the data distribution. Interactions were evaluated and stratification models were reported accordingly. Complete case analyses were performed for all assessments.

Unadjusted associations between eligible confounders to be included in the multivariable analysis including exposure variable, and the outcome were assessed using univariable binary logistic regression. The independence of NRS-HA as a prognostic factor of non-success (yes/no) was evaluated using multivariable binary logistic regression (enter), adjusting for confounders identified by DAG “base” model. An additional analysis was performed incorporating additional adjustment for education identified in the DAG socio-biological model. The same multivariable analyses were repeated using mixed logistic regression (random effects). Ordinal variables (ASA grade, duration of symptoms, education level and anxiety and/or depression) showing a linear association in univariable analysis were included in the multivariable analysis as continuous variables for better model performance, impact and reducing dichotomization (Sekula et al., 2025). None of the continuous variables were dichotomized except number of levels operated due to low rate of levels operated exceeding 2 (0.3%).

To evaluate the robustness of the associations, we stratified the best fitted model across subgroups with different (1) type of pathology, i.e. nerve root compression from bony spurs/ligaments (recess/foraminal stenosis) versus soft disc herniation, and (2) the extent of pathology (single or more level surgery) as sensitivity analysis.

Statistical analyses were performed using Statistical Package for the Social Sciences (SPSS, Version 29.0, Armonk, NY: IBM, Corp) (IBM Corp, 2023). Mixed logistic regression was performed using STATA (StataCorp, 2023).

### 2.12 Ethics

Written informed consent, including the purpose of this study, had been provided by the patients recorded in the NORspine. Approval of the study protocol was approved by the Regional committee for medical and health research ethics North-Norway (REK Nord ref: 753118).

## 3 Results

Mean age (SD) was 49.8 (9.4) years and 2879 (46.2%) were women. At 12 months follow-up 1848/6234 (29.6%) did not respond to the questionnaire. Detailed differences between responders and non-responders have been published previously (Habiba et al., 2026).

### 3.1 Patients baseline characteristics

Patients in the non-success group had similar mean age as the success group (51.2), had more comorbidity (ASA grade >2, 8.3% vs 6.3%, p = 0.026), and were more likely to be smokers (28.9% vs 18.8%; p < 0.001). In the non-success group, a lower proportion worked full time (30.4% vs 39.7%; p < 0.001) and had University or college education (32% vs 44.9%; p < 0.001). They also reported longer duration of head/neck symptoms, pain and disability at baseline (Table 1).

**Table 1:** Baseline characteristics of patients with neck disability index (NDI) improvement of ≥35% (success) and <35% (Non-success) 12 months after surgery for degenerative cervical radiculopathy.

	Total study population N = 3618	NDI change ≥35% (Success) N = 2240	NDI change <35% (Non-success) N = 1378	P-value^j^
Age, years; mean (SD)^a^	51.2 (9.1)	51.2 (9.0)	51.2 (9.1)	0.898
Gender; n (%)				
Male	1955 (54.0)	1231 (55%)	724 (52.5)	0.157
Marital status: n (%)				
Single	809 (22.4)	461 (20.6)	348 (25.3)	0.003
Cohabitant	719 (19.9)	450 (20.1)	269 (19.5)	
Married	2077 (57.4)	1323 (59.1)	754 (54.7)	
Missing	11 (0.3)	4 (0.2)	7 (0.5)	
Body mass Index, mean				
mean (SD)	27.4 (4.4)	27.3 (4.2)	27.6 (4.6)	0.075
Normal (0-25), n (%)	1136 (32)	729 (33)	407 (30.3)	0.115
Overweight (25-30), n (%)	1545 (43.5)	953 (43.2)	592 (44)	
Obesity class I^b^, n (%)	690 (19.4)	419 (19)	271 (20.1)	
Obesity class II^c^, n (%)	147 (4.1)	91 (4.1)	56 (4.2)	
Obesity class III^d^, n (%)	34 (1.0)	15 (0.7)	19 (1.4)	
Smokers; n (%)				
Yes	808 (22.7)	415 (18.8)	393 (28.9)	<0.001
Missing	54 (1.5)	35 (1.6)	19 (1.4)	
Level of education; n (%)				
Primary and secondary education	443 (12.3)	221 (9.9)	222 (16.1)	<0.001
Vocational school	1226 (33.9)	704 (31.5)	522 (37.9)	
High school	470 (13)	290 (13)	180 (13.1)	
University or college education <4 years	816 (22.6)	546 (24.4)	270 (19.6)	
University or college education ≥4 years	607 (16.8)	443 (19.8)	164 (11.9)	
Missing	54 (1.5)	34 (1.5)	20 (1.5)	
University or college education				
Yes, n (%)	1423 (39.9)	989 (44.9)	434 (32.0)	<0.001
Work status; n (%)				
Working full time	1309 (36.2)	890 (39.7)	419 (30.4)	<0.001
Homeworkers	18 (0.5)	12 (0.5)	6 (0.4)	
Students	12 (0.3)	8 (0.4)	4 (0.3)	
Age pension	184 (5.1)	120 (5.4)	64 (4.6)	
Unemployed	36 (1.4)	13 (0.6)	23 (1.7)	
Sick leave	1360 (37.6)	876 (39.1)	484 (35.1)	
Active sick leave	104 (2.9)	70 (3.1)	34 (2.5)	
Rehabilitation	235 (6.5)	74 (3.3)	161 (11.7)	
Disability pension	296 (8.2)	142 (6.3)	154 (11.2)	
Disability pension and sick leave	39 (1.1)	22 (1.0)	17 (1.2)	
Missing	24 (0.7)	12 (0.5)	12 (0.9)	
Receiving sickness/Disability benefits Yes, n (%)	2284 (63.6)	1337 (60.0)	947 (69.3)	<0.001
ASA grade^e^^,^ n (%)				
I	1164 (32.2)	831 (37.1)	333 (24.2)	<0.001
II	2138 (59.1)	1236 (55.2)	902 (65.5)	
III	251 (6.9)	139 (6.2)	112 (8.1)	
IV	0 (0.0)	0 (0.0)	0 (0.0)	
V	0 (0.0)	0 (0.0)	0 (0.0)	
Missing	65 (1.8)	34 (1.5)	31 (2.2)	
ASA grade > II, n (%)	251 (7.1)	139 (6.3)	112 (8.3)	0.026
Duration of head or neck pain; n (%)				
No neck or head pain	98 (2.7)	67 (3.0)	31 (2.2)	<0.001
Less than 3 months	379 (10.5)	319 (14.2)	60 (4.4)	
3 to 12 months	1099 (30.4)	782 (34.9)	317 (23.0)	
1 to 2 years	595 (16.4)	353 (15.8)	242 (17.6)	
More than 2 years	1393 (38.5)	688 (30.7)	705 (51.2)	
Missing	54 (1.5)	31 (1.4)	23 (1.7)	
Duration of arm pain; n (%)				
None	76 (2.1)	40 (1.8)	36 (2.6)	<0.001
Less than 3 months	508 (14.0)	420 (18.8)	88 (6.4)	
3 to 12 months	1379 (38.1)	936 (41.8)	443 (32.2)	
1 to 2 years	696 (19.2)	386 (17.2)	310 (22.5)	
More than 2 years	889 (24.6)	426 (19.0)	463 (33.6)	
Missing	69 (1.9)	32 (1.4)	37 (2.7)	
NRS^h^ head, mean (SD)	3.7 (3.0)	3.2 (2.9)	4.5 (2.9)	0.668
NRS neck, mean (SD)	6.0 (2.4)	5.8 (2.5)	6.3 (2.3)	0.015
NRS arm, mean (SD)	6.3 (2.4)	6.3 (2.4)	6.2 (2.3)	0.189
NDI^i^ total score, mean (SD)	39.7 (14.6)	39.1 (14.4)	40.6 (14.7)	0.298
Procedure and morphology, n (%)				
ACDF^f^	3590 (99.2)	2232 (99.6)	1371 (95.5)	0.493
With plate	42 (1.2)	18 (0.8)	24 (1.7)	0.011
ADR^g^	28 (0.8)	12 (0.5)	16 (1.2)	0.133
Disc herniation/soft disc	2534 (70)	1648 (73.6)	886 (64.3)	<0.001
Spondylosis/bony spurs	1084 (30)	592 (26.4)	492 (35.7)	<0.001
Levels operated: n (%)				
More than one level	1160 (32.2)	651 (29.1)	509 (37.1)	<0.001
Missing	0 (0.0)	0 (0.0)	0 (0.0)	
Length of hospital stay (days), mean (SD)	1.59 (1.5)	1.46 (1.5)	1.81 (1.6)	0.353
Work related posture, n (%)				
Work with arms over shoulder level	381 (10.5)	222 (9.9)	159 (11.5)	<0.001
Mostly computer work	1134 (31.3)	798 (35.6)	336 (24.4)	
Hard physical labour	815 (22.5)	447 (20.0)	368 (26.7)	
Slight physical labour with a variety of positions	656 (18.1)	444 (19.8)	212 (15.4)	
Mainly seated at work	189 (5.2)	119 (5.3)	70 (5.1)	
Missing	443 (12.2)	210 (9.4)	233 (16.9)	

a Standard deviation.

b Class I (30-35).

c Class II (35-40).

d Class III >40.

e American Society of Anesthesiologists.

f Anterior cervical discectomy and fusion.

g Artificial disc replacement.

h Numeric rating scale.

i Neck disability index.

j Chi square and independent samples t-tests.

## 4 Outcomes

The overall success rate after for DCR surgery at 12 months in complete cases was 2240/3618 (62%). The reductions in NDI, NRS-AP and NRS-NP after 12 months were noticeably less in the DCR-HA group compared to the group without headache (Table 2).

**Table 2:** Complete case analyses of patient reported outcome measures among subgroups with degenerative cervical radiculopathy (DCR) associated headache and those without headache (HA).

Outcome	Subgroup	Baseline	12 months follow-up	Mean change
NRS^a^-NP^b^: mean (95% CI^c^)	DCR-HA	6.45 (6.4 – 6.5)	3.5 (3.4-3.6)	2.9 (2.8 – 3.0)
	No DCR-HA	4.46 (4.3-4.6)	1.8 (1.7-2.0)	2.6 (2.4-2.8)
NRS-AP^d^: mean (95% CI)	DCR-HA	6.4 (6.32-6.45	3.0 (2.9 – 3.11)	3.38 (3.3 – 3.5)
	No DCR-HA	5.95 (5.8-6.01	1.72 (1.6-1.9	4.25 (4.0-4.5)
NDI^e^: mean (95% CI)	DCR-HA	42.7 (42.3-43-1)	24.7 (24.0-25.3)	17.6 (16.9-18.2)
	No DCR-HA	32.3 (31.5-33.2)	12.6 (11.7-13.6)	19.5 (18.3-20.6)

a Numeric rating scale.

b Neck pain.

c Confidence interval.

d Arm pain.

e Neck disability index.

### 4.1 Association of headache and non-success

**Fig. 2:**
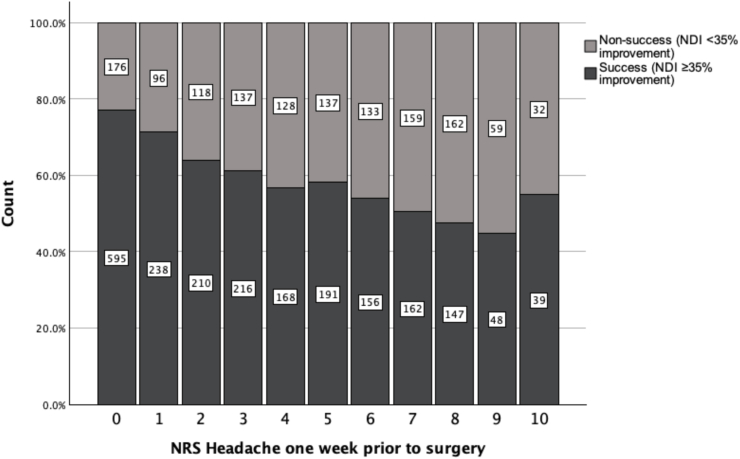
Preoperative headache severity (Numeric rating scale (NRS), 0-10) at baseline by outcome categories of success and non-success, according to neck disability index (NDI) improvement.

In univariate analysis NRS-HA had a significant association to non-success at 12 months (NDI<35%) with a 15% increase in odds ratio for each one unit increase of baseline NRS-HA (OR:1.15, 95% CI: 1.13-1.18, p-value <0.001), as illustrated in Fig. 2 and Supplementary Table 2.

Based on the DAG-evaluation, the following covariates were included in the multivariable “base” model: Age, sex, ASA grade, anxiety, smoking status, duration of neck/head symptoms, headache-and arm pain intensity (NRS: 0-10), as well as extent (single- or multi-level) and type (disc herniation or lateral stenosis due to spondylosis) of spinal pathology (Supplementary Table 1).

**Fig. 3:**
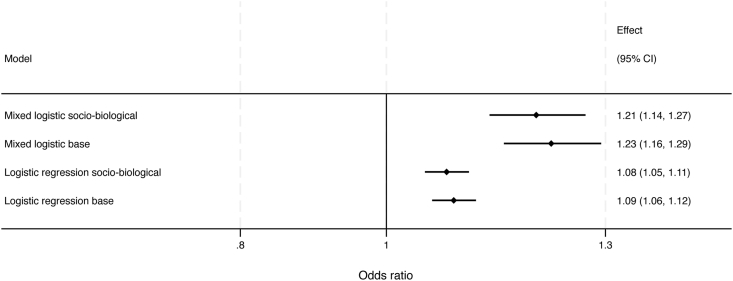
Different multivariable models assessing the association (odds ratio) between preoperative severity NRS-Headache and non-successful outcomes of surgery for degenerative cervical radiculopathy.

The exposure and confounders were included in complete case-and mixed binary logistic regression models. The socio-biological model showed the best model fit (LR χ2 (1) = 31.77, p < 0.001) and is therefore presented as main result (Table 3). Higher education was associated with less likelihood of non-success (OR 0.84, 95% CI 0.79–0.89). Adjusted for the confounders, an NRS-HA increase by one point at baseline increased the odds ratio by 8% non-success at 12 months follow up (OR: 1.08, 95% CI: 1.05-1.11, p < 0.001) in complete case analyses and by 21% (OR 1.21, 95%CI: 1.14-1.27, p < 0.001) in the mixed logistic regression model (Table 3), as illustrated in Fig. 3. The corresponding figures for baseline NRS-HA in the base model was a 9 % of non-success (OR:1.09, 95% CI: 1.06-1.12, p < 0.001) in complete case- and 23% (OR:1.23, 95% CI: 1.16-1.29, p < 0.001) in the mixed logistic regression model (Supplementary Table 2).

**Table 3:** Multivariable analyses with and without random (mixed model) effects assessing the independent association of degenerative cervical radiculopathy (DCR) associated headache (HA) on Neck disability index (NDI) non-success (<35%, yes/no), adjusted for possible confounding factors. (Final model “Sociobiological”).

Variables	Multivariable analyses N = 3212		Multivariable analyses with random effects N = 6662	
	**Odds ratio (95% CI)**^c^	**p-value**	**Odds ratio (95% CI)**	**p-value**
NRS^a^-HA^b^	**1.08** (1.05-1.11)	0.001	**1.21** (1.14-1.27)	<0.001
Age	0.99 (0.98 – 1.00)	0.009	0.97 (0.96 – 0.99)	<0.001
Female	1.05 (0.89 – 1.22)	0.562	1.37 (1.06-1.76)	0.015
Smoking (yes/no)	1.30 (1.08 –1.56)	0.005	1.27 (0.95 – 1.71)	0.108
Anxiety^d^	1.44 (1.26 – 1.64)	<0.001	2.48 (1.91 – 3.20)	<0.001
Duration of neck and head symptoms	1.38 (1.29–1.49)	<0.001	1.77 (1.57-2.00)	<0.001
NRS-AP	0.95 (0.91-0.98)	0.001	0.85 (0.85-0.81)	<0.001
Two or more level surgery	1.23 (1.04 – 1.45)	0.013	1.61 (1.24-2.11)	<0.001
Bony spurs/spondylosis	1.24 (1.05-1.47)	0.012	1.62 (1.22-2.13)	0.001
ASA^e^	1.39 (1.21-1.61)	0.005	1.82 (1.44-2.30)	<0.001
Level of education	0.84 (0.79-0.89)	<0.001	0.75 (0.68-0.83)	<0.001

a Numeric rating scale.

b Headache.

c Confidence interval.

d EuroQol-five dimension three level “Anxiety/depression level, moderate to severe”.

e American anestesiology score (I-V).

The sensitivity analysis showed that our main finding was robust across patient groups with different type and extent of cervical spine pathology i.e. NRS-HA remained an independent prognostic factor for non-success (Supplementary Table 3).

## 5 Discussion

We found that more headache at baseline was an independent prognostic factor of a non-successful outcome, in terms of persistent neck pain-related disability, even if headache tends to improve postoperatively along with symptoms of neck- and arm pain. The odds ratio increased by approximately 20% per point increase in baseline NRS-HA according to the best fitted models. The sensitivity analyses showed that the results were robust across subgroups with different types and amount of cervical spine pathology. The findings were consistent in the complete case analyses, but the association between NRS-HA and the outcome was somewhat weaker. To the best of our knowledge, this is the first study showing this association.

However, patients previously operated in the cervical spine, those operated for other conditions, and patients undergoing more complex procedures were not included in this study. We did not include patients operated with posterior foraminotomy to evaluate headache as a potential prognostic factor in a homogenous cohort. The rationale was that posterior muscle dissection and retraction, could increase axial neck pain and related headache at short term, compared to anterior surgery. However, a recent study by Svensson et al. with 2 years follow up reported that posterior decompression was associated with slightly more headache improvement (Svensson et al., 2025).

The pathophysiology of DCR-HA is poorly understood and the definitions used in the literature are inconsistent, making comparison of results across studies difficult (Persson et al., 2007; Thind et al., 2020; Sun et al., 2015; Schrot et al., 2014; Zhou et al., 2024; Yang et al., 2022; Pang et al., 2020; Shimohata et al., 2017; Liu et al., 2016; Riina et al., 2009). Importantly, the aim of our study was not to explore the etiology or to improve headache classification, but to evaluate NRS-HA as a prognostic factor, irrespective of is origin. Such information can be of value in individual based health care, especially for modelling accurate probability estimates for different patient outcomes.

### 5.1 Strengths and limitations

This study represents a large sample of patients from real world clinical practice, being operated with the most common cervical procedure. This would indicate high clinical relevance and external validity. We have used well validated PROM's showing results which coincide well with outcome data from surveys and registries in the US and other countries (American Spine Registry, the quality outcome database and Swespine). (Khan et al., 2020; Devin et al., 2021; Svensson et al., 2025; Tabanli and Eren, 2024).

Despite the large sample size this study has some weaknesses. The most important is possible unmeasured confounders which might affect assessments, of both exposure and outcome. For instance, we know from previous studies that comorbidities tend to be underreported in spine registries (Alhaug et al., 2022; Ingebrigtsen et al., 2023). We excluded patients with other headache diagnoses as recorded by a physician at baseline. In the NORspine, previous headache problems being reported under “other comorbidity” were not specified further and headache medication (i.e for migraine) was not recorded (Habiba et al., 2026). Therefore, we cannot rule out that some cases with DCR-headache might have been misclassified as other comorbidity.

Diagnosis and radiological findings were reported by the treating surgeons. We have not tested intra -and inter-rater reliability of the surgeons’ assessments. Thus, misclassification might exist.

In the present study, the follow-up rate was 70%. According to prior NORspine reports, these data can be regarded as missing at random (MAR) (Ingebrigtsen et al., 2023). We applied mixed logistic regression to account for missing data, utilizing all available longitudinal observations to ensure unbiased estimates under the MAR assumption (Twisk et al., 2013). Additional imputation was not considered necessary.

### 5.2 Implications and future perspectives

DCR-HA may indicate underlying musculoskeletal disorders and more chronic and complex neck pain problems. Accordingly, our results lend support to previous recommendations that dominance of headache symptoms warrants surgical restraint (Persson et al., 2007; Thind et al., 2020). This should be communicated clearly to DCR patients, and referral to multidisciplinary clinical evaluation and/or non-surgical treatment can be advisable. In many countries, incidence rates of DCR surgery have increased (Neifert et al., 2020; Kristiansen et al., 2016). Being challenged by aging populations, new knowledge about prognostic factors is needed to improve patient selection for surgery and patients’ expectations about treatment outcomes (Taso et al., 2020, 2022). Still, further studies are needed to classify DCR-HA and evaluate if it is a genuine treatment effect modifier, or if the DCR-headache itself can be modified/improved prior to surgery by other treatment modalities.

## 6 Conclusion

Higher preoperative headache severity was an independent prognostic factor for non-successful outcomes following DCR surgery. This information can be used in preoperative counselling, aimed at improving patient selection and prognosis.

## 7 Previous presentations

The abstract was accepted for a rapid-fire presentation at the Global Spine Congress, which took place in Rio de Janeiro, Brazil, on May 29, 2025.

## Funding

This work was supported by the 10.13039/501100007137Northern Norway Regional Health Authority [grant numbers HNF1717-24].

## Declaration of competing interest

The authors declare the following financial interests/personal relationships which may be considered as potential competing interests:

Samer Haiba reports was provided by Northern Norway Regional Health Authority research fund. If there are other authors, they declare that they have no known competing financial interests or personal relationships that could have appeared to influence the work reported in this paper.

## Figures and Tables

**figs1:**