# Supplementary material for: Preoperative headache severity and the risk of unsuccessful outcomes after anterior surgery for degenerative cervical radiculopathy: a population-based study from the Norwegian Registry for Spine Surgery
Source: Brain Spine. 2026 May 16;6:106100. doi: 10.1016/j.bas.2026.106100 (PMC13213792; doi:10.1016/j.bas.2026.106100)
Supplement: Multimedia component 1 [file mmc1.docx]

| **Supplementary Table 1:** Description of variables included in DAG and the univariable and multivariable analyses. | | | |
| --- | --- | --- | --- |
| **Variables** | **Description of variable property in univariable analysis and DAG** | **Description of variable property in multivariable analysis** | **base or socio-biological model variable by DAG** |
| Age | Age in years at time of surgery. Numeric/continuous | Continuous | base |
| Sex | Male/female. | Nominal | base |
| Educational level | Ordinal variable:   1. Primary and secondary education 2. Vocational school 3. High school 4. University or college education < 4 years 5. University or college education ≥ 4 years   As there was a linear relationship in univariable analysis, the variable was included in the multivariable analysis as a continuous variable without dichotomization. | Ordinal used as continuous | Socio-biological |
| Smoking | Yes/no. | Nominal | Base |
| NRS-NP^a^ | Pain scale from 0 (no pain) to 10 (worst conceivable pain). Numeric/continuous | Not included in multivariable analysis. | Base |
| NRS-AP^b^ | Pain scale from 0 (no pain) to 10 (worst conceivable pain). Numeric/continuous | Continuous | Base |
| ASA grade^c^ | The variable is ordinal and categorized as:   1. ASA 1 2. ASA 2 3. ASA 3 4. ASA 4 5. ASA 5.   As there was a linear relationship in univariable analysis, the variable was included in the multivariable analysis as a continuous variable without dichotomization. | Ordinal used as continuous | Base |
| Body mass index (BMI) | Reported weight and hight are used for calculating the BMI at baseline. Continuous. | Not included in multivariable analysis. | Base |
| EQ-5D 3L anxiety and/or depression^e^ | Ordinal:   1. Not anxious or depressed 2. Moderately anxious or depressed 3. Severely anxious or depressed.   Continuous. | Ordinal used as continuous. | Base |
| Duration of neck pain | The response categories in the NORspine questionnaire are:   1. I have no pain 2. Less than 3 months 3. 3 to 12 months 4. 1-2 years 5. More than 2 years.   This variable showed a linear association in univariable regression, it was included in the multivariable regression as a continuous variable. | Ordinal used as continuous | Base |
| Duration of arm pain | The response categories in the NORspine questionnaire are (Ordinal variable):   1. I have no pain 2. Less than 3 months 3. 3 to 12 months 4. 1-2 years 5. More than 2 years. | Not included in multivariable analysis. | Base |
| Number of levels opererated | Reported in the registry as a continuous variable (numeric). Dichotomized to one vs several level surgery (yes/no) as the number of cases 3 level or more amount to less the 0.3%. | Nominal | Base |
| Spondylotic changes/bony spurs | Nominal variable, based on a categorization of the radiological findings checked by the surgeon. Spondylotic changes (yes) and prolaps (no) | Nominal | Base |
| Desk job/Office work/Work that is not physically strenuous | Nominal variable reported as:   1. Work with arms over shoulder level 2. Mostly computer work 3. Hard physical labour 4. Slight physical labour with a variety of positions 5. Mainly seated at work   Dichotomized to office work (yes (2,5)/no (1,3-4). | Nominal | Base |
| Work status | Nominal, reported as the following by the registry:   1. Full time work 2. Homeworkers 3. Students 4. Age pension 5. Unemployed 6. Sick leave 7. Active sick leave 8. Rehabilitation 9. Disability pension 10. Disability pension and sick leave   Dichotomized to receiving sickness benefits yes (2-10) and no (1) | Not included in multivariable analysis. | Socio-biological |
| ^a^Numeric rating scale for neck pain. ^b^Numeric rating scale for arm pain. ^c^American Society of Anesthesiologists.^d^EuroQol-five dimension 5^th^ item three level | | | |

| **Supplementary Table 2:** Univariable-, multivariable and random effects (mixed model) assessing degenerative cervical radiculopathy (DCR) associated headache (HA) as a prognostic factor of non-success Neck disability index (NDI) improvement <35%, adjusted for possible confounding factors (Base model). | | | | | | |
| --- | --- | --- | --- | --- | --- | --- |
| **Variables** | **Univariable analysis** | | **Multivariable analysis**  **N=3259** | | **Multivariable analysis with random effects**  **N=6761** | |
|  | **Odds ratio (95% CI)** ^e^ | **p-value** | **Odds ratio (95% CI)** | **p-value** | **Odds ratio (95% CI)** | **p-value** |
| Age | 1.00 (0.99 – 1.01) | 0.898 | 0.99 (0.98 – 1.00) | 0.014 | 0.97 (0.85-0.95) | 0.001 |
| Female | 1.10 (0.96 – 1.26) | 0.157 | 0.99 (0.84 – 1.15) | 0.861 | 1.22 (0.95-1.56) | 0.114 |
| Smoking (no/yes) | 1.75 (1.50 – 2.06) | <0.001 | 1.46 (1.22 – 1.74) | <0.001 | 1.56 (1.18-2.09) | 0.002 |
| Body mass index | 1..01 (1.0 – 1.03) | 0.098 |  |  |  |  |
| Bony spurs (no/yes) | 1.55 (1.34 – 1.79) | <0.001 | 1.23 (1.04 – 1.46) | 0.017 | 1.64 (1.24-2.16) | 0.001 |
| Duration of neck and head symptoms  <3 month  3 months - 12 months  1 year – 2 years  >2 years | 0.41 (0.24-0.68)  0.87 (0.56-1.37)  1.48 (0.94-2.34)  2.21 (1.43-3.43) | 0.001  0.561  0.09  <0.001 | 1.39 (1.29-1.49) | <0.001 | 1.80 (1.60-2.03) | <0.001 |
| Desk job/Office work/Work that is not physically strenuous^b^ (no/yes) | 0.67 (0.57 – 0.77) | <0.001 |  |  |  |  |
| Duration of arm pain  <3 months  3 months - 12 months  1 year – 2 years  >2 years | 0.23 (0.14-0.39)  0.53 (0.33-0.84)  0.89 (0.56-1.43)  1.21 (0.76-1.93) | <0.001  0.007  0.638  0.431 |  |  |  |  |
| NRS^a^-NP^d^ | 1.08 (1.05-1.11) | <0.001 |  |  |  |  |
| NRS-AP^c^ | 0.98 (0.95-1.01) | 0.226 | 0.95 (0.92-0.98) | 0.002 | 0.85 (0.81-0.90) | <0.001 |
| NRS-HP^b^ | 1.15 (1.13-1.18) | <0.001 | **1.09 (1.06-1.12)** | <0.001 | **1.23 (1.16-1.29)** | <0.001 |
| Two or more level surgery | 1.43 (1.24 – 1.65) | <0.001 | 1.25 (1.07 – 1.47) | 0.006 | 1.61 (1.24-2.09) | <0.001 |
| ASA^f^  ASA grade II  ASA grade III | 1.82 (1.56-2.12)  2.01 (1.52-2.66) | <0.001  <0.001 | 1.44 (1.25-1.67) | <0.001 | 1.91 (1.51-2.40) | <0.001 |
| Anxiety^g^  I am a moderately anxious or depressed  I’m severly anxious or  depressed | 1.78 (1.54-2.05)  2.32 (1.70-3.18) | <0.001  <0.001 | 1.46 (1.28-1.66) | <0.001 | 2.15 (1.72-2.67) | <0.001 |
| Level of education; n (%)  Vocational school  High school  University or college  education <4 years  University or college  education ≥4 years | 0.74 (0.59-0.92)  0.62 (0.48-0.80)  0.49 (0.28-0.48)  0.37 (0.28-0.48) | 0.006  <0.001  <0.001  <0.001 |  |  |  |  |
| ^a^Numeric rating scale. ^b^Headache ^c^Arm pain ^d^Neck pain ^e^Confidence interval. ^f^American anestesiology score (I-IV). ^g^ EuroQol-five dimension 5^th^ item three level “Anxiety/depression moderate to severe” | | | | | | |

| **Supplementary Table 3.** Sensitivity analysis: Multivariable and random effects (mixed model) assessing degenerative cervical radiculopathy (DCR) associated headache (HA) as a prognostic factor of non-success Neck disability index (NDI) improvement <35%, across patient groups with different type and extent of cervical spine pathology, adjusted for possible confounding factors (Socio-biological). | | | | | | |
| --- | --- | --- | --- | --- | --- | --- |
| **Variables** | **Multivariable analysis** | | | **Multivariable analysis with random effects** | | |
|  | **Odds ratio (95% CI)** ^c^ | **p-value** | **Complete cases, n** | **Odds ratio (95% CI)** | **p-value** | **Observations, n** |
| NRS^a^-HA^b^ (Multilevel surgery) | 1..06 (1.01– 1.11) | 0.018 | 1043 | 1.17 (1.07 – 1.30) | 0.001 | 2148 |
| NRS-HA (Single level surgery) | 1.09 (1.05 – 1.13) | <0.001 | 2169 | 1.22 (1.14 – 1.30) | <0.001 | 4514 |
| NRS-HA (Bony spurs/spondylosis) | 1.09 (1.03 – 1.14) | 0.001 | 952 | 1.18 (0.07 – 1.30) | 0.001 | 1944 |
| NRS-HA (Disc herniation) | 1.08 (1.04 – 1.11) | <0.001 | 2260 | 1.21 (1.13 – 1.30) | <0.001 | 4718 |
| ^a^Numeric rating scale. ^b^Headache. ^c^Confidence interval. | | | | | | |
