# Supplementary figures and images for: Preoperative headache severity and the risk of unsuccessful outcomes after anterior surgery for degenerative cervical radiculopathy: a population-based study from the Norwegian Registry for Spine Surgery
Source: Brain Spine. 2026 May 16;6:106100. doi: 10.1016/j.bas.2026.106100 (PMC13213792; doi:10.1016/j.bas.2026.106100)

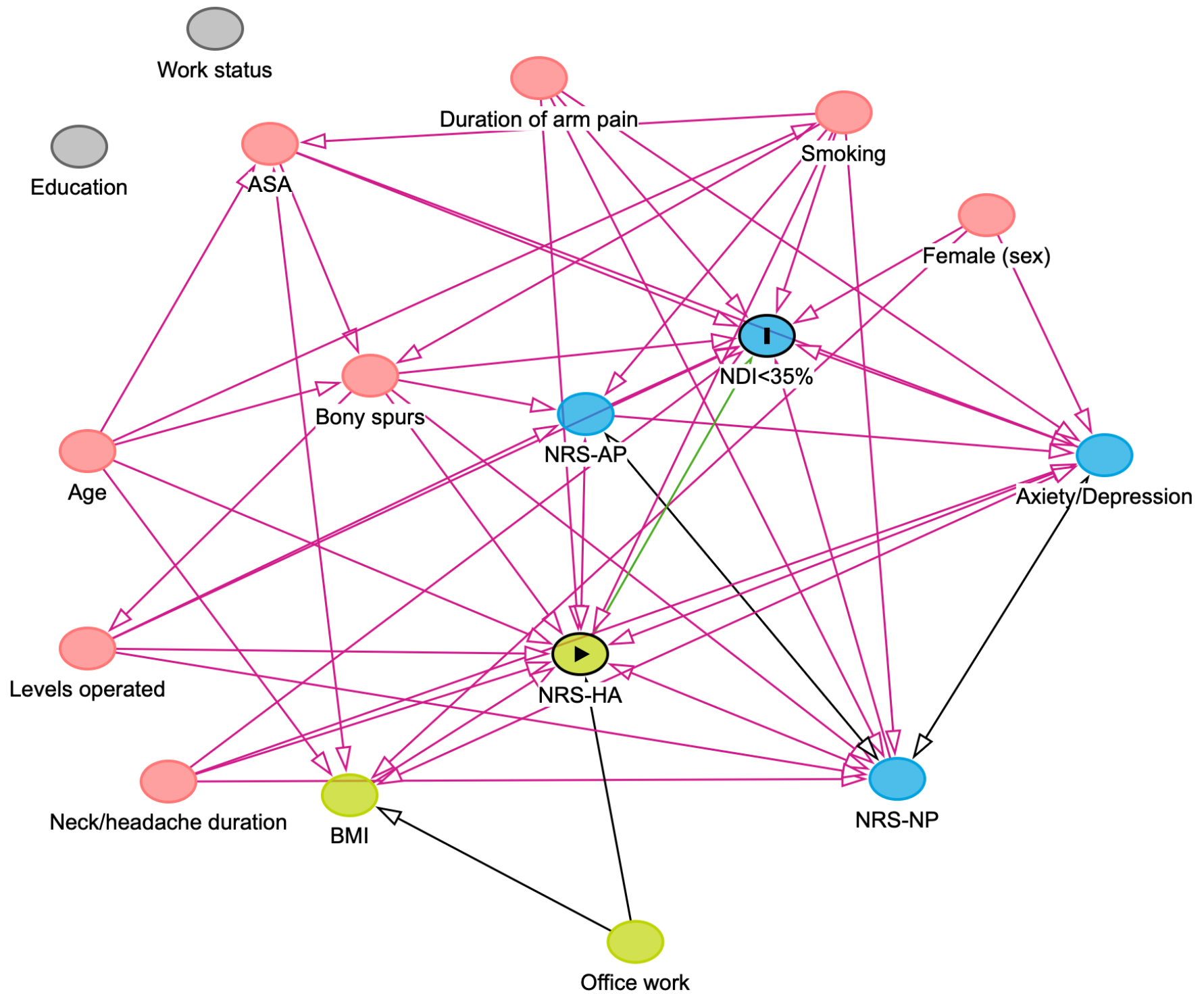

Supplement: Multimedia component 2 [file mmc2.pdf]
